# Female Participation in Academic European Neurosurgery—A Cross-Sectional Analysis

**DOI:** 10.3390/brainsci11070834

**Published:** 2021-06-23

**Authors:** Catharina Conzen, Karlijn Hakvoort, Hans Clusmann, Anke Höllig

**Affiliations:** Department of Neurosurgery, University Hospital RWTH Aachen, 52074 Aachen, Germany; cconzen@ukaachen.de (C.C.); khakvoort@ukaachen.de (K.H.); hclusmann@ukaachen.de (H.C.)

**Keywords:** neurosurgery, gender disparity, female participation, structural bias

## Abstract

The study aims to provide data on authors’ gender distribution with special attention on publications from Europe. Articles (October 2019–March 2020) published in three representative neurosurgical journals (*Acta Neurochirurgica, Journal of Neurosurgery, Neurosurgery*) were analyzed with regard to female participation. Out of 648 publications, 503 original articles were analyzed: 17.5% (*n* = 670) of the 3.821 authors were female, with 15.7% (*n* = 79) females as first and 9.5% (*n* = 48) as last authors. The lowest ratio of female first and last authors was seen in original articles published in the *JNS* (12.3%/7.7% vs. *Neurosurgery* 14.9%/10.6% and *Acta* 23.0/11.5%). Articles originated in Europe made up 29.8% (female author ratio 21.1% (*n* = 226)). Female first authorship was seen in 20.7% and last authorship in 10.7% (15.3% and 7.3% were affiliated to a neurosurgical department). The percentages of female authorship were lower if non-original articles (*n* = 145) were analyzed (11.7% first/4.8% last authorships). Female participation in editorial boards was 8.0%. Considering the percentages of European female neurosurgeons, the current data are proportional. However, the lack of female last authors, the discrepancy regarding non-original articles and the composition of the editorial boards indicate that there still is a structural underrepresentation and that females are limited in achieving powerful positions.

## 1. Introduction

During the last decades, the number of female medical students worldwide have constantly risen. Latest data from the UK for the academic year 2018/19 present a 57% female ratio in medical students [1] and numbers for other countries, e.g., Germany, are even higher (61.7%) [2]. Nevertheless, underrepresentation of women within the medical field is still evident; medical leadership positions lack female participation [3,4] and women are still underrepresented in academic publications [5,6]. Even up to now, there are indications that women within the medical field receive lower salaries compared to men [7,8,9].

Although numbers have risen during the last decades, the ratio of female authors has plateaued within the last years [5,10]. Especially female last authors and invited single authors are scarce [6,7,11] indicating that these numbers may not only arise from a male numerical superiority in some specialties. Irrespective of the reasons, female academic underrepresentation may have multiple undesirable consequences. First, the rising amount of young and ambitious female physicians lack adequate role models, particularly in surgical specialties [12,13]. Further, the filling of senior positions within medical departments and filling of editorial boards, conference speakers and leadership positions—dependent on academic visibility—even further strengthens the inequity, thus, reinforcing the existing power structure [3,9,14,15]. Lastly, female underrepresentation can result in research biases [16,17,18,19,20]; this may be due to an unconscious bias (the lack of a female perspective), the historical preference of male animals for experimental studies or, considering clinical trials, unequal inclusion of patients.

Neurosurgery in particular is a specialty with various historical and cultural obstacles for female surgeons [21]. A recent survey addressing members of the European Association of Neurosurgical Societies—EANS—“…did not imply an overall feeling of gender inequality among European respondents…” [22]. Further, the study provided data on the proportion of female neurosurgeons in Europe resulting in an overall rate of 11% female board certified neurosurgeons [22]. In contrary to the report from Steklacova and colleagues, a very up-to-date survey from the UK demonstrates that perceived discrimination is an everyday phenomenon for female neurosurgeons [23]. Further, another current study reported that the female participation at the conferences of the EANS is lower than expected and that particularly invited talks are male dominated [24].

That said, it is important to acquire as much objective data as possible to provide a basis for further discussion and subsequent measures. Although there are limitations of the following approach, such as the fact that the data are not provided according to age (assuming an increase in female neurosurgeons over time), this study aims to asses a current status quo of female academic participation focused on Europe (respectively the journal affiliated to the EANS until 2020), to discuss possible specialty-specific problems, to compare the data with the corresponding journals from the US and develop suggestions for further measures. Therefore, publications (October 2019–March 2020) of the official neurosurgical journal of the European Association of Neurosurgical Societies (*Acta Neurochirurgica*) and of the American Association of Neurosurgery, respectively, the Congress of Neurological Surgeons (with their affiliated journals *JNS* and *Neurosurgery*) were analyzed with respect to female overall contribution, first and last authorship. Further, data are evaluated according to subspecialty and origin and lastly, the composition of the editorial boards is related with the results.

## 2. Materials and Methods

This observational analysis included original articles, commentaries and letters that were published in the *Journal of Neurosurgery (JNS)*, *Neurosurgery* and *Acta Neurochirurgica* (further on referred to as *Acta*) between October 2019 and March 2020. Book reviews and in replies were excluded. Moreover, papers with unknown sex of first author and/or last author, and papers with unknown sex of >33% of all authors were excluded. Only authors with known sex were included in our analysis.

In order to determine sex, first names were checked in the online database *Genderize*, accessible via https://api.genderize.io/?name=, accessed on June 2020–December 2020. This database, created by Caspar Strømgen in 2013, makes a gender prediction based on a continuously growing dataset derived from continuous scanning of public profiles in major social networks [25,26]. By filling out a name in the URL, this database shows the most likely sex with a probability and a count. In cases with a probability of ≥0.90 and count of ≥500, we assumed the proposed gender to be correct. In cases in which this result was disputable, authors were additionally looked up on the internet, in the following order: on the website of the corresponding department, social media accounts such as research gate, LinkedIn, Xing, or via a search engine (pictures, online publications, etc.). First and last authors were always looked up on the internet additionally. In cases of remaining unclarity, the corresponding author was contacted.

For geographical distribution analyses, affiliation of the last author was set as origin (continent and country) of the article. Furthermore, data were collected in respect to the specialization or affiliated institution of each first and last author. Articles were categorized by the following twelve main topics: vascular, neuro-oncology, trauma, spine, pediatrics, functional, peripheral nerves, neurocritical care, radiosurgery, neuroanatomy, CSF circulation, and other.

### Statistical Analysis

The data were collected in Microsoft Excel (Microsoft Corporation, Redmond, WA, USA). Differences between two groups were analyzed using the two-sided Student’s *t*-test. Categorical variables were analyzed in contingency tables using the Chi-square test resp. Fisher’s exact test.

Results are reported by *p*-values. For all comparisons, alpha level for statistical significance was set to 0.05. All analyses were performed using GraphPad Prism^®^ version 9.0.2 (GraphPad Software, Inc., La Jolla, CA, USA) as well as jamovi (Version 1.2).

## 3. Results

### 3.1. Analyzed Article Types

Data of 681 articles were analyzed. Because of insufficient information on the sex of authors of 33 articles (4.7%), these were excluded (of which 72.7% (*n* = 24) had a Chinese and 15.2% (*n* = 5) a Korean last author). The corresponding authors in question were contacted in order to find out the sex distribution of the authors, but the response rate was low (<15%). The remaining 648 articles consisted of 503 (77.6%) original articles and 145 (22.4%) commentaries, letters and invited articles.

### 3.2. Overall Female Author Ratio

**Figure 1:**
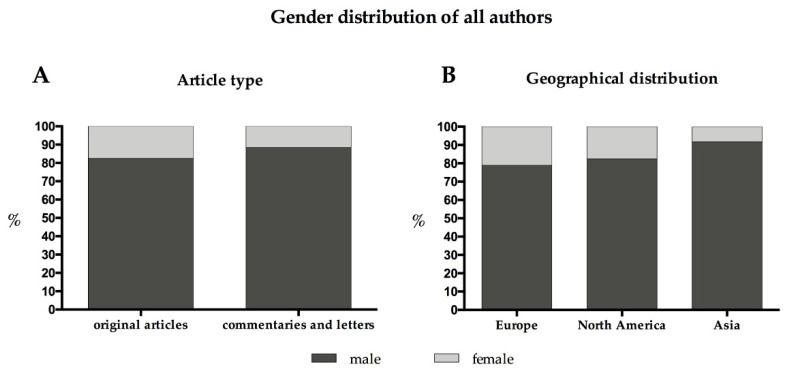
Gender distribution of all authors: (**A**) by article type and (**B**) by geographical distribution.

Of the 503 original articles analyzed, a total of 3.821 authors were counted, of whom 17.5% were female (*n* = 670) and less than half of them (47.3%, *n* = 317) were affiliated to a neurosurgical department. In 33.8% of the original articles, there was no female contribution at all, and in 75.9% (*n* = 382) both the first and last author were male (Figure 1).

**Figure 2:**
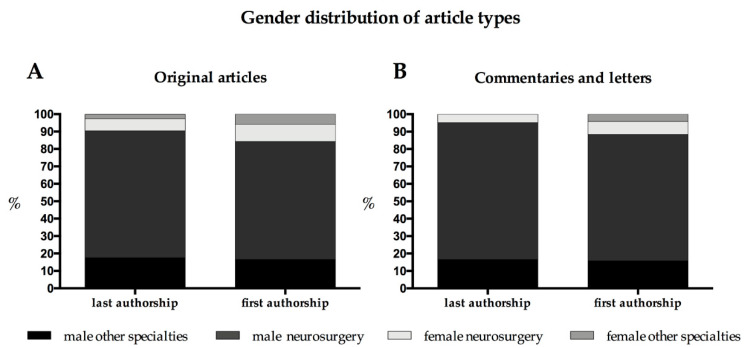
Gender distribution with regard to article type distinguishing authors affiliated to a neurosurgical department and those not affiliated to a neurosurgical department; (**A**) original articles; (**B**) commentaries and letters.

In 15.7% (*n* = 79), a female first author was found, whereas a female last author was seen in 9.5% *(n* = 48). Considering only women affiliated to a neurosurgical department, the numbers were reduced to 9.9% (*n* = 50) and 6.8% (*n* = 34) respectively. Related to the corresponding male first and last authors affiliated to a neurosurgical department, females made up 12.8% of the neurosurgically affiliated first and 8.5% of the last authors (Figure 2).

Affiliation to a neurosurgical department did not differ significantly between men and women with regard to first authorship (80.7% and 70.8% respectively, *p* = 0.218; Chi-square), but did so with regard to the last authorship (80.4% and 63.3% respectively, *p* = 0.0042).

Interestingly, papers with female contribution had a significantly higher author number: 8.7 authors compared to 5.7 authors in papers without female contribution (*p* = 0.000039, Chi-square).

### 3.3. Female Author Ratios per Journal

The lowest ratio of female first and last authors was seen in original articles published in the *JNS* (12.3% and 7.7%, respectively), followed by *Neurosurgery* (14.9% and 10.6%) and *Acta* (23.0 and 11.5%). The *JNS* also demonstrated the lowest percentage of female authors (15.8% of 1684 authors) compared to *Acta* and *Neurosurgery* (with 17.5% of 730, and 19.6% of 1407 authors). However, *Acta* had the highest percentage of original articles without female contribution: a total of 40.2% of the original articles published by *Acta* had only male authors (compared to 34.5% of the *JNS* and 27.3% of the *Neurosurgery* articles).

### 3.4. Journals’ Specifics

#### 3.4.1. Acta Neurochirurgica

The majority of the articles published (October 2019–March 2020) came from Germany (*n* = 28; 23.0%), followed by the USA (*n* = 20; 16.4%) and Switzerland (*n* = 8; 6.6%). The journal covered all neurosurgical topics; most articles (*n* = 29; 23.8%) dealt with neurooncological issues, followed by vascular (*n* = 24; 19.7%) and spine topics (*n* = 22; 18.0%). Of note, *Acta* is the journal analyzed with the lowest impact factor (2019: 1.817).

#### 3.4.2. JNS

The impact factor of the *JNS* in 2019 was 3.968. Under the umbrella of the *JNS*, there is also a spine and a pediatric section (*JNS Spine, JNS Pediatric*) as well as *Neurosurgical focus.* Since we only present results acquired from analyses of the proper *JNS*, it is plausible that the spine and pediatric sector are underrepresented in our analysis. The dominant topics identified were vascular (*n* = 51; 23.2%), neurooncological (*n* = 50; 22.7%) and functional (*n* = 40; 18.2%) ones. The articles mainly originated from the US (*n* = 124; 56.4%), followed by Japan (*n* = 21; 9.5%) and China (*n* = 14; 6.4%).

#### 3.4.3. Neurosurgery

*Neurosurgery* is currently the journal with the highest impact factor (4.853). The dominant topics were neurooncology *(n* = 33; 20.5%) and cerebrovascular issues (*n* = 28; 17.4%). Topics not classified by our scheme made up 18.6% of the articles (*n* = 30). Origin was mainly the US (*n* = 114; 70.8%), followed by Canada (*n* = 7; 4.3%) and Germany (*n* = 6; 3.7%).

### 3.5. Articles from Europe

The sum of articles (from all of the three journals) originated in Europe made up 29.8% (*n* = 150 out of 503) of the entire cohort. A total of 1073 authors was counted, with a female authors ratio of 21.1% (*n* = 226), of which *n* = 124 (11.6% of the total cohort) were affiliated to a neurosurgical department. Female first authorship was seen in 20.7% (*n* = 31); female first authorship affiliated to a neurosurgical department made up 15.3% (*n* = 23). The ratio for female last authorship was 10.7% (7.3% if only female last authors affiliated to a neurosurgical department were considered). Most European articles came from Germany (*n* = 42; 28%), followed by France (*n* = 16; 10.7%) and the UK (*n* = 15; 10%). The most common subtopics were neurooncology (*n* = 41; 27.3%; female first authorship: *n* = 8; 19.5%, female last authorship: *n* = 4; 9.8%), cerebrovascular (*n* = 28; 18.7%; female first authorship: *n* = 4; 14.3%; female last authorship: *n* = 4; 14.3%) and spine *(n* = 16; 10.7%; female first authorship: *n* = 7; 43.8%; female last authorship: *n* = 1; 6.3%).

With regard to the specific countries, the following ratios for first and last authorships were observed (specified for the most frequent countries); articles from Germany (*n* = 42) showed a female first authorship of 16.7% (*n* = 7) and last authorship of 11.9% (*n* = 5). The second most common country of origin was France (*n* = 16); female first authorship was 18.8% (*n* = 3) and last authorship was 6.3% (*n* = 1). In a total of 15 articles originating from the UK, female first authorship accounted for 26.7% (*n* = 4) and last authorship for 13.3% (*n* = 2). The details for the specific countries are summed up in Table 1.

### 3.6. Comparisons with Other Continents

The majority of original articles originated in North America (275 out of 503; 54.7%): female authors accounted for 17.5% of the authors (382 out of 2179); female first authorship was 14.2% (*n* = 39; with female neurosurgical affiliation: *n* = 25; 9.1%) and last authorship was 9.8% (*n* = 27; affiliated to neurosurgical department: *n* = 19; 6.9%). Relating only to authors affiliated to a neurosurgical department, neurosurgically affiliated females accounted for 18.0% of the first and 8.6% of the last authors of articles from Europe, 11.4% and 9.0% for articles from North America and 4.2% and 8% for articles from Asia. Again, the most frequent subtopic was neurooncology (*n* = 59; 21.5%) with a female first author ratio of 15.3% (*n* = 9) and a last authorship ratio of 5.1% (*n* = 3) followed cerebrovascular articles (*n* = 51; 18.5%) with seven articles first authored by females (13.7%) and eight articles last authored by females (15.7%).

**Figure 3:**
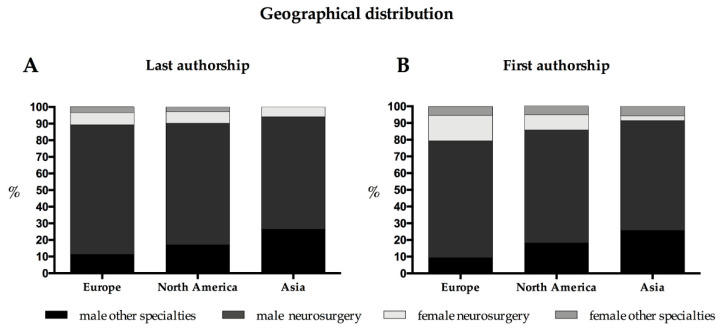
Geographical distribution of the authors: (**A**): last authorship; (**B**): first authorship.

Articles from Europe (see Section 3.5.) were second most frequent, followed by articles from Asia (n = 68; 13.5%) showing a female contribution of 8.2% (41 out of 501 authors) with a female first authorship of 8.8% (*n* = 6) and last authorship of 5.9% (*n* = 4). Of note, 29 articles from Asia were not available for analysis as it was not possible to identify the sex of the authors. The geographical distribution of female participation per continent is depicted in Figure 3.

With respect to affiliation to a neurosurgical department, neither ratios for the first nor last authorships differed significantly comparing female with male authors (each for Europe, North America and Asia).

### 3.7. Commentaries or Letters and Invited Publications

A total of 145 non original articles (comments, letters, invited publications; summing up 22.4% of all of the publications screened) were counted with a total author number of 425 (49 of these females, 11.5%; 27 of female authors affiliated to a neurosurgical department, 6.4%). Female first authors made up 11.7% (*n* = 17), whereas 7.6% of the first authors were females affiliated to a neurosurgical department. Female last authors represented 4.8% of the total last authors (all of the female last authors were affiliated to a neurosurgical department; 4.8%). The numbers of female authors were substantially lower compared to that of original articles (11.5% vs. 17.5%; *p* = 0.007; Chi-square). Female first authorship was seen in 11.7% and female last authorship in 4.8%. Both numbers were not significantly lower than the female author ratios assessed for original articles (*p* = 0.301, *p* = 0.095; Chi-square). Of note, 82.8% (120 out of 145) of the publications did not show any female participation at all.

Regarding the specific journals, *Acta* and *Neurosurgery* showed high ratios of non-original articles (28.2 and 27.8%), whereas this ratio was substantially lower for the *JNS* (13.7%).

With the exception of female first authorships in *Neurosurgery* (16.1%), all of the ratios for female first and last authorships were lower than those for original articles.

### 3.8. Editorial Boards

**Figure 4:**
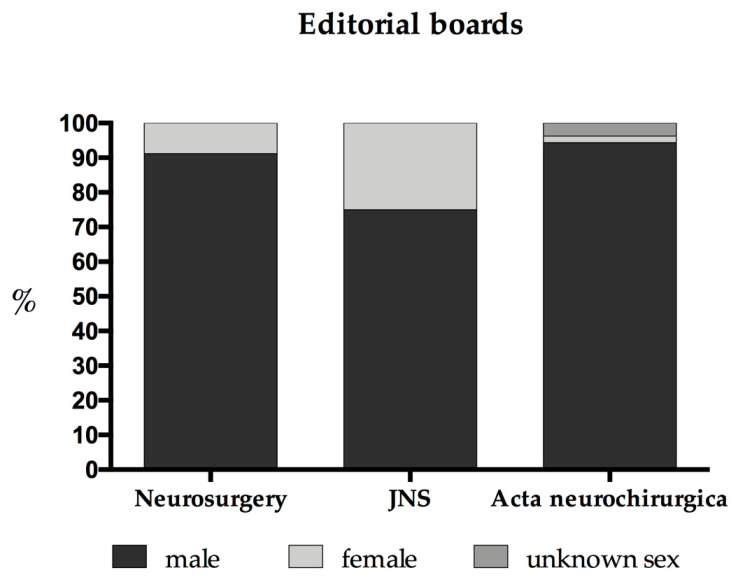
Composition of editorial boards.

The specific editorial boards may be regarded as the final organs of policymaking. Thus, the composition of each editorial board was documented additionally. All editors in chief of the surveyed journals are male; *Acta* and the *JNS* present so-called “associate editors” and “co-chairs”, of which all are male (*Acta*: six out of six, *JNS*: four out of four). There is 1 female editor out of 53 in the editorial board of *Acta* (with two editors whose sex we were not able to determine) and 0 female editors out of 30 in the advisory board. The *JNS* editorial board (without including the topic specific issues such as *JNS Spine*, etc.) includes 6 women out of 19 editors (31.6%). The editorial board of *Neurosurgery* is structured differently: The editorial advisory board consists of 2 females out of 60 editors (3.3%) and 1 female international advisory editor out of 81 (1.2%). There is also a Neurosurgery Editorial Review Board structured by topics: out of a total of 140 members 20 are female (14.3%), whereas all of the section editors (*n* = 16) are male. Summing up all of the members of the editorial boards of the journals surveyed, we find a female participation of 8.0% (*Neurosurgery*: 23 out of 260 (8.1%); *Acta*: 1 out of 90 with two persons of unknown sex (1.1%); *JNS*: 6 out of 24 (25%)) (see Figure 4).

## 4. Discussion

After analysis of all original articles published in the *JNS*, *Neurosurgery* and *Acta* *Neurochirurgica* during a six-month period (October 2019 and March 2020), an average percentage of female authors was found in 17.5%. In 33.8% of the original articles, there was no female contribution at all. Particularly last authors rarely were female. The sum of all articles originated in Europe made up 29.8% of the entire cohort. A total of 1073 authors was counted showing a female author percentage of 21.1%, of which 11.6% of the total cohort were affiliated to a neurosurgical department.

Thus, we were able to underline previous data on female participation published in 2020 by Aslan and colleagues [27], who analyzed authorships in two neurosurgical journals (*JNS* and *Neurosurgery*) between 2003 and 2018. An increase in female first authorship was seen over time (12% to 16.5%) [27]. However, ratio of female last authorship remained low (11.7% in 2003 vs. 10.5% in 2018) [27]. Here, we report even lower ratios for female participation (15.7% first authorship vs. 9.6% last authorship). Interestingly, the latest report by Pastor-Cabeza and colleagues not only subcategorized authors affiliated to a neurosurgical department, but also the corresponding degree, thus, aiming to distinguish between neurosurgeons and other people such as basic scientists affiliated to a neurosurgical department [6]. According to this definition, only 3.6% of the last authors were female neurosurgeons. Thus, a more in-depth analysis of our data might have revealed even lower percentages of female participation.

Of note, there are differences between the specific journals with the *JNS* showing the lowest numbers both for female first and last authorship (12.3% and 7.7%), with numbers for female last authorships being even lower than in 2003 (at that time 13%) [27]), whereas *Acta* scored highest (23.0 and 11.5%). However, the impact factor of *Acta* trails the ones of the *JNS* and *Neurosurgery* distinctly (1.817 vs. 3.968 and 4.853 for 2019). Further, female authorship in *Acta* seems to be clustered, as almost half of the articles published (40.2%) have no female participation at all.

With regard to the time trend of female authorship, after an increase female authorships seem to plateau, as already pointed out by others [5,10]. Of note, by using custom R scripts, Holman and colleagues generated data on around 30 million articles (PubMed (2002–2016) and arXiv indexed), indicating the author gender ratios (first, last and total authorship) dependent on journal, topic and year of publication. Interpolating the acquired courses (https://lukeholman.github.io/genderGap/, accessed on June 2020–December 2020) estimated values may be retrieved for dates after 2016 (year of publication). With the exception of *Neurosurgery*, the journals have not reached the interpolated female author ratios (*JNS*: 15.8 vs. estimated 20.2%, *Acta*: 17.5 vs. estimated 21.4%). *Neurosurgery* outperformed its estimated ratio of 18.4% by 1.2% (real female overall participation: 19.6%). However, with exception of first authorships in *Acta* (23.0 vs. estimated 20.2%), no journal has reached the estimated values for female first or last authorship, thus, underscoring the phenomenon of plateaued author gender ratios.

Significantly less authors of North-American articles were female compared to authors of European articles (17.5% vs. 21.1%), and they were significantly less often affiliated to a neurosurgical department. Asian articles had the lowest level of female contribution (8.2%), showing the lowest percentage of female first and last authorship (8.8% and 5.9%) and the lowest percentage of affiliation to a neurosurgical department in female authors (3.5%; even though all four senior authors were affiliated to a neurosurgical department). It should be noted, however, that a substantial number of Asian articles (31.7%) had to be excluded due to insufficient data on the sex of the authors. For this reason, especially Chinese papers are underrepresented in our analysis (3.6% of original articles).

For Europe, an overall ratio of 12% for female neurosurgeons is reported [22]. Thus, the observed female authorship ratio of 11.5% for female authors affiliated to a neurosurgical department may reflect the aforementioned proportion quite well. Taking only the authors affiliated to a neurosurgical department into account, the percentage of female first authorship is even higher (18%) and female last authorships make up 8.6%. Thus, a general underrepresentation is not seen and the academic performance of female neurosurgeons in Europe at least seems to correspond to the actual amount of female neurosurgeons. Same applies for the US, where about 9% of the neurosurgeons are female (data for 2020; personal communication AANS). Thus, a female authorship ratio affiliated to a neurosurgical department (articles from the US only) of 8.1% may seem plausible. This is particularly true, if only authors affiliated to a neurosurgical department were taken into account, resulting in 11.4% female first and 9.0% last authorships for articles from North America. However, it has to be taken into account that affiliation to a neurosurgical department not necessarily means that only neurosurgeons or neurosurgical residents were regarded. As mentioned earlier, Pastor-Cabeza and colleagues clearly pointed out that a more extensive analysis may reveal that the real percentages of female neurosurgeons as authors may be far lower [6].

In general, the percentages of female authors decrease substantially irrespective from the origin of the article, as it comes to first and especially last authorships. Further, particularly non-original articles (such as comments, letters and invited publications) show a male preponderance. The latter could be influenced by biased preferences of the largely male editorial offices [28,29]. With exception of the *JNS*, the editorial boards are over proportionally male dominated (each 1.2% female editors for *Acta* and *Neurosurgery*). Moreover, Holman and colleagues estimated in 2016 by a databank analysis that men receive twice as many invitations from journals [7]. Further, the vicious circle of “lower position and lower performance” may contribute [30]. This hypothesis is supported by the fact that females are invited less frequently as speakers to conferences [31]. It also has to be taken into account that some aspects may be due to so-called “self-selection of women into clinical careers” [29]. However, this may be a response to due multifactorial problems and reasons for these patterns still have to be elucidated.

There are two additional facts depicted by our data: First, female contribution was associated with higher number of authors. This finding is in line with data from Parish and colleagues demonstrating that male authors predispose for lower collaboration [32]. However, we did not analyze this specific finding more extensively.

Second, we observed that *Acta*, representing the region with the highest female ratio, has the lowest percentage of female editorial board members. It is difficult to explain this finding. The—in comparison to Europe—earlier formation of a female organization within the US neurosurgical society (WINS—women in neurosurgery), which celebrated its 30th anniversary in 2020, may have contributed to an earlier awareness of the necessity and added profit of female participation [33].

### 4.1. Causes for Female Underrepresentation and Strategies for Its Increase

Considering the low female neurosurgeon ratios (e.g., 12% in Europe), in general females are underrepresented in neurosurgery. A huge number of causes may contribute to this fact; some will be discussed in detail:

First, neurosurgery is historically a male dominated subspecialty. Still, few women choose (or are chosen for) a neurosurgical residency [34]. The lack of female role models in leadership positions strengthens this pattern and influences career choices more than the classical lifestyle issues [35,36]. Second, pay disparity and sexual harassment are still prevalent in the medical sector resulting in restrain towards certain employments and specialties. As it comes to payment, the situation is very heterogeneous, as salaries and the processes of generating salary vary substantially between countries. However, some strategies apply for the entirety; in a recent article by Woodhams and colleagues [37] seven recommendations are mentioned, among them being the increase in transparency of the gender pay gaps, addressing structural barriers, implementing programs for robust analysis of gender gaps, review of the clinical performance and adjustment of salary, and making senior jobs accessible for women. Third, socially accepted family friendly employment models for both men and women are essential. Still, preferentially women—voluntarily or forced by necessity—reduce working hours due to family needs [38]. Employment models lack flexibility, the opportunity for paternal leave (for both mothers and fathers), family friendly schedules and child care support. Further, especially fathers claiming parental leave experience constraints due to lack of cultural acceptance [39]. Fourth, the dominance of males as editors and reviewers [40] may create a peer effect resulting in a preference for male authors in prominent positions (e.g., last authorship, invited authorship). Each final decision may be influenced by the specific personal background, which not necessarily has to be a conscious process. This may lead to a peer effect resulting in preference for articles by male authors. However, data are conflicting; Squazzoni and colleagues recently pointed out, that manuscripts authored or coauthored by women sometimes even are favored [41]. In contrast, studies have shown that double blind peer review actually increases diversity and female participation [42,43]. Anyway, double blind peer review represents the most objective way of performing a review and, thus, should be regarded as the “Gold-Standard”.

### 4.2. Limitations and Future Research

An important limitation of this study may be caused by the process of online research: to define the gender of the contributing authors, the sex of the given name was determined via a large online database. This may entail several biases. First, although there was a high probability of defining the correct sex via the online database, we cannot preclude that especially seldom given names were incorrectly allocated, thus, somehow skewing our data. To minimize this bias, an additional extensive online research via social media, homepages of departments and personal contact to the corresponding author was performed in case of inconclusive data.

Second, there is a bias because of underrepresentation of certain cultural area. In particular, Asian authors had to be excluded due to inconclusive given names (often used for both sexes), missing representation in free accessible social media and homepages of departments only available in the local language. Nevertheless, as the Asian papers determined a comparatively small proportion in the analyzed journals, we postulate that the conclusions supported by our data are feasible at least for the western cultural areas. Third, the definition of sex by the given name and, in inconclusive cases, by social media, etc., may neglect the fact, that gender and sex could differ in single cases. It has to be mentioned, that gender issues that are addressed in this paper are probably not the only disparity issues within neurosurgical research. There might be other disparities in academic and clinical neurosurgery that are unaccounted for in this paper, e.g., ethnicity, country of origin and sexual orientation, that are—of course—unwanted and can result in research biases. In the future, these other aspects of diversity need to be addressed as well. Moreover, reasons and possible solutions for the occurrence of disparities need to be further assessed.

Moreover, we assessed “neurosurgical affiliation” only for male first and last authors. The overall percentages, thus, do not distinguish between male authors affiliated to a neurosurgical department or not. Additionally, affiliation to a neurosurgical department does not equal being a neurosurgeon. Thus, we were not able to distinguish between neurosurgeons and other people affiliated to a neurosurgical department (such as statisticians, psychologists, other scientists…).

It could not be detected if a “generation gap” may contribute to different percentages of female participation as the authors’ age was not assessed.

The presented period of time analyzed is short and, therefore, sample size is low. Thus, especially partial results subcategorizing the cohort have to be interpreted carefully. Therefore, also general percentages (such as total female vs. total male authors) were given.

Lastly, female representation may change over time and, therefore, authors’ age may be advisable to asses. However, it would be difficult to decide if data should be related to first/last or mean authors´ age. It may rather depend on the scientific ambiance and maybe the specific working group culture, which may largely be up to the individual group leader.

Further, it is important to emphasize that a so-called “grievance culture” is destructive and that the efforts should be focused on detecting (historical, unconscious, cultural…) biases by objective means and creating a fair and constructive workspace allowing the best performance possible.

## 5. Conclusions

Considering the percentages of European female neurosurgeons, the current data are proportional, particularly if only authors affiliated to a neurosurgical department (but which is not be equalized with neurosurgeons per se) were taken into account. However, the lack of female last authors, the discrepancy regarding non-original articles and the composition of the editorial boards indicate that there still is a structural underrepresentation and that females are limited in achieving powerful positions. To put it bluntly, females in European neurosurgery do make a contribution to the academic performance according to their quantity, but the rewarding positions are still male dominated. Thus, females—with an appropriate scientific performance—should demand such positions and the neurosurgical community should stimulate them in order to weaken historically created structures and allow a just and performance driven allocation of positions.

To gain further insights, a more in-depth analysis, including age, specific field of work and a larger sample size is necessary.

## Figures and Tables

**Table 1:** Details for female participation dependent on the specific European country in alphabetical order. Female authorships are listed as a percentage of total authors. Articles with female first and last authorships are listed as percentage of the number of articles of the corresponding counting.

Country	Number of Articles	Total Authors	FEMALE AUTHORSHIPS NR./%		FEMALE FIRST AUTHORSHIPS NR./%		FEMALE LAST AUTHORSHIPS NR./%	
**AUSTRIA**	6	42	9	21.4%	1	16.7%	1	16.7%
**BELGIUM**	2	15	1	6.7%	0	0%	0	0%
**Czech Republic**	2	10	1	10.0%	0	0%	1	50.0%
**Denmark**	5	23	4	17.4%	1	20.0%	1	20.0%
**Finland**	6	35	10	28.5%	4	66.7%	0	0%
**France**	16	167	39	23.4%	3	18.7%	1	6.3%
**Germany**	42	285	62	21.8%	7	16.7%	5	11.9%
**Greece**	2	15	0	0%	0	0%	0	0%
**Italy**	9	76	18	23.7%	3	33.3%	1	11.1%
**Netherlands**	12	78	18	23.1%	2	16.7%	3	25.0%
**Norway**	6	30	4	13.3%	0	0%	1	16.7%
**Poland**	2	13	1	7.7%	0	0%	0	0%
**Portugal**	1	4	1	25.0%	1	100%	0	0%
**Serbia**	1	8	0	0%	0	0%	0	0%
**Spain**	4	31	9	29.0%	1	25.0%	0	0%
**Sweden**	5	28	2	7.1%	0	0%	0	0%
**Switzerland**	14	116	21	18.1%	4	28.6%	0	0%
**UK**	15	97	26	26.8%	4	26.7%	2	13.3%
